# Empowering people living with HIV (PLHIV): unveiling care gaps and identifying opportunities for improving care for PLHIV in Singapore and Hong Kong

**DOI:** 10.1002/jia2.26250

**Published:** 2024-05-10

**Authors:** Chen Seong Wong, Andrew Chidgey, Kai Lung Lee, Phoenix K. H. Mo, Timothy Wong, Sumita Banerjee, Vanessa Ho, Yangfa Leow, Regina Gowindah, Ying Jie Yew, Ricky Fung, Agnes Lau

**Affiliations:** ^1^ National Centre for Infectious Diseases Singapore Singapore; ^2^ Department of Infectious Diseases Tan Tock Seng Hospital Singapore Singapore; ^3^ Yong Loo Lin School of Medicine National University of Singapore Singapore Singapore; ^4^ AIDS Concern Hong Kong SAR Hong Kong SAR; ^5^ Centre for Health Behaviours Research The School of Public Health and Primary Care The Chinese University of Hong Kong Hong Kong SAR Hong Kong SAR; ^6^ Hong Kong Coalition of AIDS Service Organisations Hong Kong SAR Hong Kong SAR; ^7^ Hong Kong AIDS Foundation Hong Kong SAR Hong Kong SAR; ^8^ Action for AIDS Singapore Singapore; ^9^ Project X Singapore Singapore; ^10^ Oogachaga Singapore Singapore; ^11^ Oracle Life Sciences Singapore Singapore; ^12^ Gilead Sciences Hong Kong SAR Hong Kong SAR

**Keywords:** people living with HIV, virological suppression, physical wellbeing, psychosocial wellbeing, patient‐physician alliance, HIV care continuum

## Abstract

**Introduction:**

This study explored the behaviours of people living with HIV in Singapore and Hong Kong in terms of achieving and maintaining their physical and psychological wellbeing in relation to HIV, to identify the challenges and support needed in HIV care.

**Methods:**

This qualitative study involved 90‐minute interviews among Singapore and Hong Kong people living with HIV aged ≥18 years to explore health‐related quality of life perceptions and gaps in patient empowerment in HIV care during February–May 2022. The COM‐B (C: Capability; O: Opportunity; M: Motivation; B: Behaviour) framework was used during data analysis to identify behaviour facilitators and barriers for people living with HIV to achieve and maintain their wellbeing. Detailed accounts of respondents’ experience of living with and managing HIV, that is what worked well, unmet needs and perceived significance of wellbeing indicators, were analysed qualitatively via a combination of inductive content and deductive frameworks.

**Results:**

A total of 30 and 28 respondents were recruited from Singapore (SG) and Hong Kong (HK), respectively. Most respondents were aged 20−49 years (SG: 83.3%; HK: 64.3%), males (SG: 96.7%; HK: 92.9%), men who have sex with men (SG: 93.3%; HK: 71.4%), had university or higher education (SG: 73.3%; HK: 50.0%) and were fully employed (SG: 73.3%; HK: 57.1%). In both Singapore and Hong Kong, physical health was considered a key focus of overall wellbeing, albeit attention to long‐term health associated with cardiovascular and renal health was less salient. The impact of symptoms, side effects of treatment, mood and sleep were among the top wellbeing indicators of importance. Respondents felt that insufficient information was provided by physicians, citing consultation time and resource constraints impeding further expression of concerns to their physicians during consultation. Respondents prioritized functional wellness and delegated psychosocial health to supportive care professionals, patient groups, families and/or friends.

**Conclusions:**

There is a need in Singapore and Hong Kong to empower people living with HIV to establish better communications with their physicians and be more involved in their treatment journey and equally prioritize their psychosocial wellbeing.

## 1 INTRODUCTION

As of 2022, about 39.0 million people were living with human immunodeficiency virus (HIV) globally, with an estimated 0.7% of people living with HIV (hereafter “PLHIV”) aged 15−49 years [1]. In 2022, Singapore reported 9331 cumulative HIV cases [2], while Hong Kong recorded 11,539 cumulative cases as of the third quarter of the same year [3].

The Joint United Nations Programme on HIV/AIDS (UNAIDS) adopted a new Global AIDS Strategy 2021−2026, setting a higher 95‐95‐95 target for 2025 to be achieved: 95% of all people living with HIV to know their status, 95% of the diagnosed to receive sustained antiretroviral therapy (ART) and 95% of those receiving antiretroviral therapy to be virally suppressed. These targets sought to bridge inequalities in treatment coverage and outcomes and accelerate HIV incidence decreases by focusing on progress geographically, in all sub‐populations and age groups [4].

Both Singapore and Hong Kong share a similar population size and ethnic distribution with a relatively low prevalence of HIV, but new cases continue to arise [5, 6]. In Singapore, the National HIV Programme is supported by the collaboration between healthcare, academicians and other industry stakeholders working towards coordinating the country's HIV response [7]; striving towards the 90‐90‐90 target with 91% of PLHIV obtaining treatment and having non‐detectable viral loads [8]. Similarly, the Hong Kong Advisory Council of AIDS has proposed 10 targets to be achieved by 2026 to guide the implementation of strategies to decrease transmission (e.g. access to condoms) [9, 10]. The “Test and treat” strategy by Hong Kong that initiates treatment immediately upon diagnosis has allowed >97% of PLHIV under public sector care to receive antiretroviral therapy.

Albeit the advances in HIV care in Singapore and Hong Kong, limitations exist locally with no comprehensive social protection in place and there remains a degree of stigma linked to HIV. Furthermore, at the point of the research, laws exposed PLHIV without commensurately protecting them from resulting harm, creating a disincentive for HIV testing, further stigmatizing HIV [8]. There is a lack of HIV‐related knowledge [11] supporting findings on the pathway between poor understanding, increased stigma and decreased intent to share one's HIV status [12]. Examining perceptions of PLHIV in Singapore and Hong Kong, especially considering their continued efforts to manage HIV, shared regional characteristics and challenges in HIV, provides valuable insights into the effectiveness of local approaches towards achieving the UNAIDS goals and strategies for effective patient status improvement.

Talks about a “fourth 90” advocating for a better quality of life (QoL), entailing attention to comorbidities and self‐perceived life quality is ongoing [13]. As a majority of PLHIV are growing older, the likelihood of additional comorbidities requiring simultaneous management exists [14]. Hence, there is a need to identify key populations, define multimorbidity and polypharmacy management, and better account for multimorbidity burden and increased life expectancy to plan future requirements for HIV individual care. Additionally, PLHIV show a lower self‐perceived QoL, particularly anxiety or depression [15], highlighting the need to expand the HIV care continuum to encompass patients’ QoL holistically. The majority of mental and social wellbeing gaps of PLHIV are centred around public and workplace stigmatization and discrimination [16, 17]. Varying stigma types faced including social factors, for example loneliness, social estrangement and limited or weakening of social networks as PLHIV grow older, attribute towards elevated poorer mental health [18, 19].

Understanding how these multi‐faceted challenges manifest through different components (from the COM‐B Framework [20]) can more accurately identify the gaps that need to be addressed. Specifically, understanding how physical and psychological capacity (Capability) such as stigma, physical and social environment (Opportunity) including infrastructural barriers, as well as emotional and evaluative processes (Motivation). By utilizing the framework, this study allowed for a rigorous understanding of how PLHIV behave in terms of achieving and maintaining their physical and psychological wellbeing in relation to HIV, to identify the challenges and support needed.

## 2 METHODS

### 2.1 Study design

This qualitative study used one‐to‐one (virtual or face‐to‐face), in‐depth interviews to explore factors influencing health‐related QoL perceptions and behaviour of PLHIV in achieving and maintaining their wellbeing in Singapore and Hong Kong.

### 2.2 Ethics approval and informed consent

The study protocol (#21‐CERN‐101) and the interview discussion guide were submitted to Pearl Pathway Institutional Review Board (IRB) (Indianapolis, USA) for exemption determination and were determined to be exempt according to FDA 21 CFR 56.104 and 45CFR46.104(b)(2): (2) Tests, Survey, Interviews.

All respondents in the study had provided informed consent and respondents’ confidentiality was assured. Only deidentified information was collected and analysed. Rights to refuse and withdraw at any time during the study were accepted.

### 2.3 Study population and recruitment

PLHIV were invited to be interviewed through an email invitation from local community‐based organizations and all interviews were conducted during February–May 2022. PLHIV respondents had to be prescribed and currently on ART for ≥12 months and had undetectable HIV viral load in the latest follow‐up.

The interview guide was developed in English and translated into local languages which were proofread by linguists who are native speakers of the languages. The 90‐minute interviews were conducted by experienced moderators in English and the local language(s) of Hong Kong (Cantonese) and Singapore (Mandarin and Malay) and were recorded and transcribed verbatim anonymously.

It included a narrative approach so that PLHIV can provide detailed accounts of their experiences of living with HIV to highlight what mattered most to them, what worked well and their unmet needs. Interviews incorporated the use of projective and enabling techniques to elucidate unconscious thoughts and feelings and more truthful answers which otherwise may not have been done through direct and structured questioning [21]. During the interviews, PLHIV were also asked about their perception of wellbeing indicators and ranked which they perceived to be most important.

### 2.4 Theoretical framework

The study utilized the COM‐B framework [20] as the overarching model to understand behaviour through three constructs: Capability (individual psychological and physical capacity), Opportunity (physical and social environment) and Motivation (emotional and evaluative processes). The framework posits that for PLHIV to live well and achieve optimal wellbeing, the three components under COM‐B need to be present. It underpinned the design of the discussion guide and was used during data analysis to identify behaviour facilitators and barriers for PLHIV to achieve and maintain their wellbeing.

### 2.5 Qualitative data analysis

Transcribed interviews were coded and analysed thematically utilizing the NVivo qualitative data analysis software. The study used a combination of inductive content analysis and deductive framework analysis through the COM‐B framework. After immersion of the data, researchers went through open coding of transcripts to capture emergent concepts. These concepts were further developed based on new transcripts and aligned with the Principal Investigator and the research team. Concepts were subsequently analysed through the COM‐B model—assessing behaviours of PLHIV based on their Capability, Opportunity and Motivation—to identify behaviour barriers and facilitators.

## 3 RESULTS

### 3.1 Study population characteristics

A total of 30 respondents were recruited from Singapore (SG) and 28 respondents from Hong Kong (HK) (Table 1). Most of the respondents were aged 20−49 years (SG: 83.3%, *n* = 25/30; HK: 64.3%, *n* = 18/28), males (SG: 96.7%, *n* = 29/30); HK: 92.9%, *n* = 26/28), men who have sex with men (SG: 93.3%, *n* = 28/30; HK: 71.4%, *n* = 20/28). At least half had university or higher education (SG: 50.0%, *n* = 15; HK: 50.0%, *n* = 14), and were employed full‐time (SG: 73.3%, *n* = 22; HK: 57.1%, *n* = 16). About one‐tenth had the average monthly income that was considered to be the ideal salary in the respective geographic regions, that is 13.3% (*n* = 4) in Singapore had a monthly income of SGD 6001−9000 [22] and 17.9% (*n* = 5) in Hong Kong had a monthly income of HKD≥30,000 [23].

**Table 1:** Demographic profile of participants from Singapore (*N* = 30) and Hong Kong (*N* = 28)

		Singapore (*N* = 30)		Hong Kong (*N* = 28)	
		*N*	%	*N*	%
**Age**	*20−49*	25	83.3%	18	64.3%
	*50−65*	5	16.7%	10	35.7%
**Sex**	*Male*	29	96.7%	26	92.9%
	*Female*	1	3.3%	2	7.1%
**Sexual orientation**	*Men who have sex with men*	28	93.3%	20	71.4%
	*Heterosexual*	2	6.7%	4	14.3%
	*Bisexual*	0	0.0%	4	14.3%
**Education level**	*Secondary*	13	43.3%	13	46.4%
	*Diploma*	2	6.7%	0	0.0%
	*University*	12	40.0%	9	32.1%
	*Post‐Grad*	3	10.0%	5	17.9%
	*Other*	0	0.0%	1	3.6%
**Employment status**	*No regular*	7	23.3%	5	17.9%
	*Part‐time*	1	3.3%	7	25.0%
	*Full‐time*	22	73.3%	16	57.1%
**Monthly income (average)** (1 USD = 1.36 SGD) (1 USD = 7.84 HKD)	*<SGD 3000 (<USD 2206)*	7	23.3%	n/a	
	*SGD 3001−6000 (USD 2207−4412)*	12	40.0%	n/a	
	*SGD 6001−9000 (USD 4413−6618)*	4	13.3%	n/a	
	*<HKD 10,000 (<USD 1276)*	n/a		8	28.6%
	*HKD 10,000−16,999 (USD 1276−2168)*	n/a		2	7.1%
	*HKD 17,000−29,999 (USD 2168−3826)*	n/a		8	28.6%
	*HKD 30,000−49,999 (USD 3827−6377)*	n/a		3	10.7%
	*≥HKD 50,000 (≥6378)*	n/a		2	7.1%
	*No regular income*	5	16.7%	3	10.7%
	*Refuse to share*	2	6.7%	2	7.1%

Abbreviation: n/a, not applicable.

Source for USD conversion (April 2022): https://www.xe.com/

**Figure 1:**
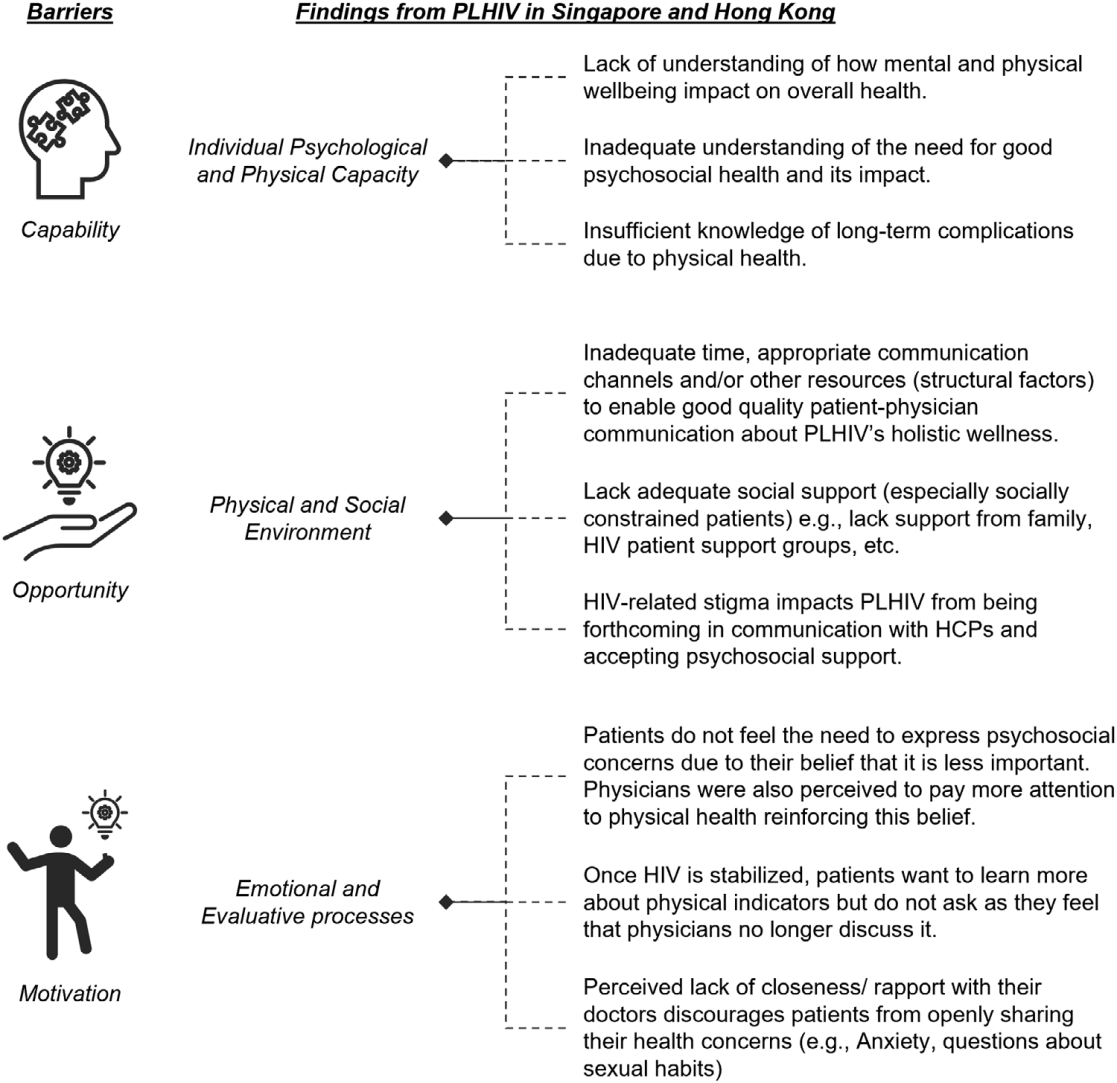
Barriers hindering PLHIV from maintaining their wellness and quality of life identified through the COM‐B framework.

Findings analysed through the COM‐B framework revealed barriers related to the capability, opportunity and motivation that can be addressed to promote behaviour changes to improve their QoL (Figure 1). Inadequacies in patient‐physician communications and alliance, and limited knowledge as well as insufficient focus on psychosocial wellness appeared to have an impact on holistic HIV health management and, eventually, treatment outcomes.

### 3.2 Perception of HIV as a manageable condition and wellbeing as a continuum

HIV was recognized by the respondents as manageable, similar to chronic conditions that have seen improved treatment and management with the ability to control symptoms and side effects. Respondents perceived that once HIV is under control with good management of their health, they could resume activities and “can basically live a normal life just like those without HIV.” Respondents had good information on self‐care, with younger respondents deferring to information searches on the Internet and older respondents relying on their PLHIV peers and support groups.

Respondents viewed good wellbeing as maintaining a healthy lifestyle and a positive mental outlook, for example “someone who knows how to take care of themselves,” experiencing little or no symptoms of HIV, has a healthy and fit body, “to be in good spirits, outgoing.” These wellness perceptions were mainly based on their personal lived experiences and observations, drawing comparisons with other chronic diseases, such as diabetes and high blood pressure. Furthermore, physicians also influenced how respondents approached wellness through regular monitoring and encouragement to adhere to medication and maintain a good physical condition.

*“… the doctor will keep giving you instructions to follow… if you have high cholesterol, then you have to follow doctor's instructions to regulate your diet. If you are willing to do it, your overall health conditions can be very good”* ***HK05, 20***−***49 y.o. Male, Men who have sex with men***

### 3.3 Knowledge (psychological capability) of physical health versus psychosocial health

#### 3.3.1 Physical health is considered a key focus of overall wellbeing

**Figure 2:**
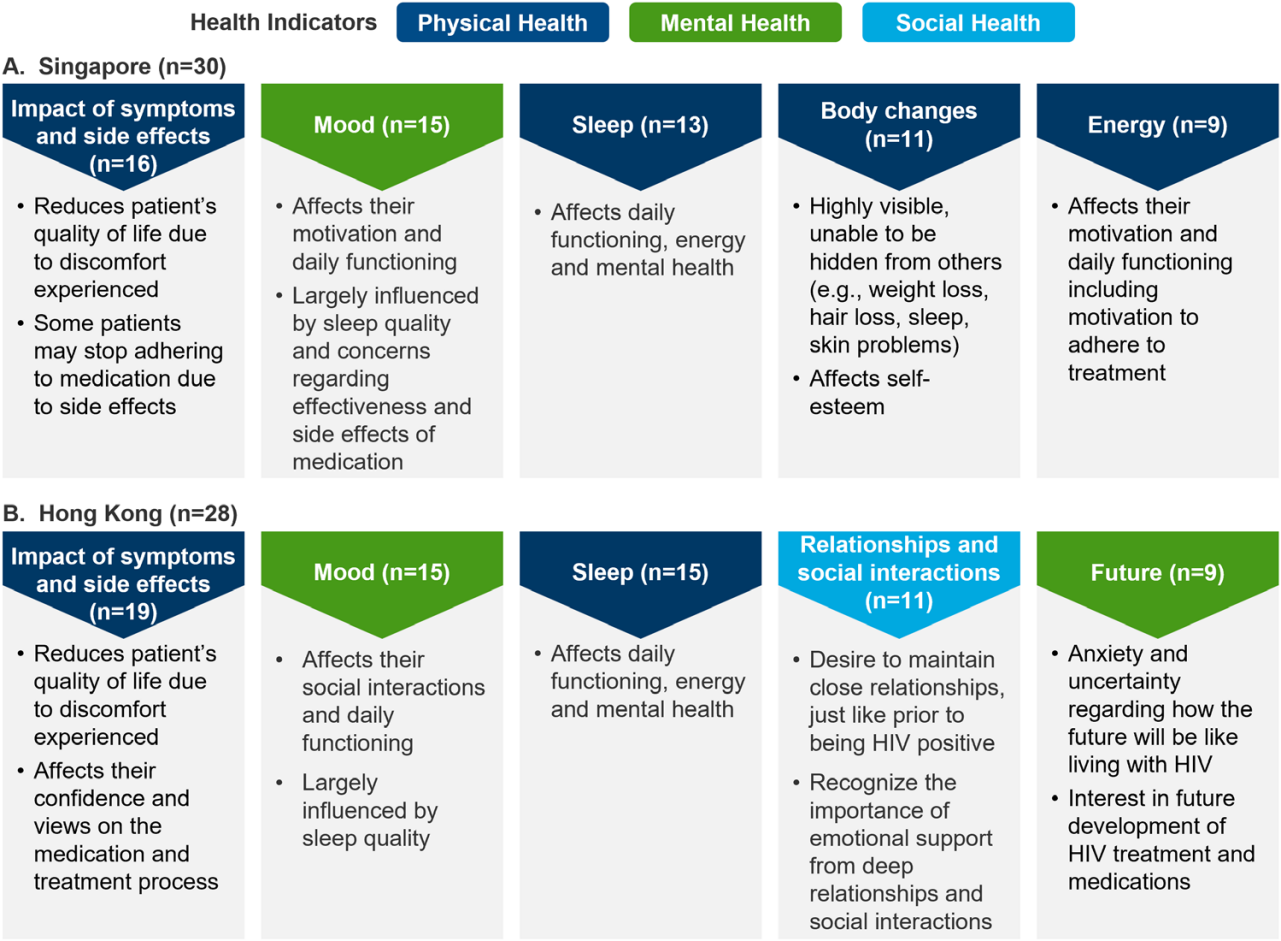
Top five wellbeing indicators perceived to be important to the respondents. Indicators perceived by respondents in Singapore (*n* = 30) and Hong Kong (*n* = 28) to be important in their wellbeing.

When respondents were asked to rank wellbeing indicators of physical, mental and social health, physical health indicators were ranked higher than mental and social health indicators. Impact of symptoms, followed by side effects, mood and sleep were among the top wellbeing indicators considered most important to PLHIV in Singapore and Hong Kong (Figure 2). In both Singapore and Hong Kong, “sleep” was commonly perceived to affect daily functioning, energy and mental health. However, some differences in the respondents’ perceptions were observed between Singapore and Hong Kong in the aspect of “mood.” For instance, respondents in Singapore identified mood with medication side effects, while those in Hong Kong identified a relationship between their mood with sleep.

Respondents shared that they had adopted healthier lifestyles to keep their bodies in check, for example weight maintenance, blood glucose and lipid levels, and taking medication as instructed, to avoid long‐term complications. Information shared by physicians during consultations was well‐received by the respondents and most were motivated to adhere to medication regimen to ensure their physical indicators were healthy. Respondents focused on their physical health because of the complications arising from HIV, bodily pain or aches and public health issues (e.g. the COVID‐19 pandemic) that could impact HIV care. They were also influenced by their physicians’ focus on HIV markers and virological suppression (i.e. through CD4 levels in Hong Kong and/or viral load in both countries).

Despite this, respondents reported feeling that they might not have adequate information regarding the maintenance of physical wellbeing, especially after their HIV has stabilized. They also seemed to place less emphasis on the long‐term dimensions of physical health, particularly in relation to cardiovascular and renal wellbeing. Respondents were aware of the check‐up procedures and health indicators after the condition is stabilized but expressed a preference for physicians to provide further explanations of the health indicators and their relevance to their health. Yet, the current clinical setting does not encourage patients to express their concerns further.

*“There are things that I wish (the doctor) will discuss to me more about how to increase my CD4… “Your CD4 is fine, your viral load is fine,” and that's it, that's all the conversation we have… there is no point for me to try very hard to increase my CD4, because it's already stuck there. There is no goal, (to) get CD4 to a certain value… this is not a conversation we are having, so I took it as it's final, it is what it is. It's something it does not feel like I can change.”* ***SG03, 20***−***49 y.o. Male, Men who have sex with men***

### 3.4 Motivation and Opportunity (environmental factors) impacting Knowledge (psychological capability)

#### 3.4.1 Psychosocial health was less prioritized in overall health and wellbeing

Psychosocial health was found to be less top of mind than physical health during the interviews with respondents. Despite recognizing the importance of both physical and psychosocial health aspects for their overall wellbeing, respondents were in practice not managing their psychological health as optimally as their physical health. Findings indicate that this behaviour could be attributed to the physician's focus on functional wellbeing during consultation and follow‐ups, while psychosocial health could have been delegated to the supportive care professionals or patient support groups, or family and friends.

Yet, subconsciously, respondents’ main motivations for the maintenance of physical health stemmed from a psychosocial need and desire to look well and live their lives like “individuals without HIV” (HIV being an immunodeficient condition which could cause one to appear unwell). Similarly, men who  sex of men and male bisexuals were motivated to keep symptoms under control to engage in social activities confidently.

*“I think physical health is more important, like the presence of skin conditions, hair loss and changes in strength.”* ***HK06, 20***−***49 y.o. Male, Bisexual***

*“…for body changes, I don't want to look sick, I don't want (to) look weak. Then, impact of symptoms or side effect, I always want less side effects.”* ***SG11, 20***−***49 y.o. Male, Men who have sex with men***

Respondents also confided that they prioritized physical health due to fear and anxiety that their psychological health would be affected due to HIV, that is feeling isolated and discriminated against.

#### 3.4.2 Multiple barriers may underlie psychological health being a priority

Respondents shared that they were reluctant to ask for help or support and did not actively manage their psychosocial health due to the stigma attached to HIV. There were also self‐reports of being in denial of their condition and not seeking professional help immediately to manage their HIV.

*“A mentally and physically stable patient will still need to face HIV stigma and discrimination, which is another barrier to overcome … HIV‐related stigma remains a key barrier for me to disclose my HIV status to my family and friends.”* ***HK11, 20***−***49 y.o. Male, Bisexual***

*“… I was very zombified for two weeks…. (Patient organization) told me to drop by if I want to and gave me an envelope. When I left, I threw away the envelope…they also gave me a good booklet…about concise information regarding…treatment and HIV. I didn't read… threw away the booklet…I threw them away, so I'm not reminded that I have (HIV)…”* ***SG20, 20***−***49 y.o. Male, Men who have sex with men***

Physicians were not expected by patients to be responsible for managing and caring for PLHIV's health beyond monitoring treatment and clinical indicators; hence, patients did not actively reach out for psychosocial support from physicians. While patients perceived therapists or psychiatrists as the main support for emotional health, a minority of respondents had engaged these professionals for psychosocial support with more reports from Hong Kong.

On top of the stigma attached to HIV, there was great reluctance among heterosexual female PLHIV to receive psychotherapeutic care and support even when their physicians had referred them. Respondents cited reasons for feeling isolated and lack of belonging given that most patient support groups focus on the lesbian, gay, bisexual, transsexual and queer community (LGBTQ).

*“Last time I say (patient support group) was only talk, no action…talk for 10 minutes, then go…. But they are trying, they do have a women's group, but it is no longer working because…(women) shy away; that is why they concentrate more on LGBT, compared to people like (married women)… for us, we don't voice out, we don't tell what we want…”* ***SG02, 20***−***49 y.o. Female, Heterosexual***

#### 3.4.3 Clinical settings do not facilitate holistic discussions on wellbeing between patients and physicians

Psychosocial health was felt to be less of a focus in the interest of consultation time or resources. The long waiting times and short consultation duration were reported by respondents in both Singapore and Hong Kong. There was insufficient time to clarify their health conditions resulting in prioritizing of physical health discussions.

Furthermore, respondents did not feel comfortable expressing feelings and thoughts related to their condition as current clinical settings were non‐conducive in facilitating a close and trusting patient‐physician alliance. In Singapore, respondents were assigned the same physician for each consultation and had a better rapport to speak more openly with their physician. However, time and resource restraints impeded adequate coverage of psychosocial wellness during consultation. In contrast, PLHIV in Hong Kong do not have an assigned attending physician due to limited HIV clinics. PLHIV were assigned to any available physician (rotation of physicians) hindering rapport and relationship building with their physicians. Instead, nurses supporting the physicians may follow up with the patient on factors associated with psychosocial health, for example understanding sexual health that could impact the patients’ physical health.

*“(it is) easy to get stressed seeing a doctor…. Seeing a different doctor every time will also affect the patient's emotion…will feel more at ease if seeing a doctor who is familiar with our conditions.”* ***HK17, 50***−***65 y.o., Male, Men who have sex with men***

The respondents also reported feeling a lack of emotional connection with their physicians and attributed this to the healthcare providers not being adequately skilled to discuss psychosocial health.

*“I think doctors try not to touch on my mental and social health, because they are not trained to, they don't want to open a can of worms and cannot close it.”* ***SG12, 50***−***65 y.o., male, Men who have sex with men***

Additionally, supportive healthcare providers (e.g. psychologists, counsellors) were perceived by some respondents as not having an adequate understanding of them as HIV patients beyond the HIV symptoms, for example how they manage their psychosocial wellness with regard to HIV (such as sexual health). They also felt somewhat stigmatized by healthcare providers. Advice from the healthcare providers was not well received and participants could not trust the healthcare professionals (HCPs) to communicate and share information or concerns with them.

*“…in Hong Kong…many of the counsellors are not HIV infected, they don't really understand how many issues an infected person is facing every day”* ***HK01, 20***−***49 years old, male, Men who have sex with men***

## 4 DISCUSSION

This study provided qualitative exploratory insights into the perceptions of living with HIV in Singapore and Hong Kong as well as current gaps in achieving a good QoL, especially in terms of psychosocial health and support. Using a narrative approach and associative and projective techniques, respondents could express their thoughts and feelings truthfully and openly and uncover subconscious thoughts and feelings about their experiences living with HIV.

From our study, there was an awareness among the respondents in both regions on the importance of achieving holistic wellbeing through the adoption of pro‐health behaviours associated with a healthier lifestyle including diet modifications, and self‐monitoring of health status through clinical indicators [24, 25, 26]. However, PLHIV's attention to long‐term aspects of physical health relating to cardiovascular and renal health appeared to be less salient. This could be indicative of a lower awareness of comorbidity risks associated with HIV and implied a possible gap in patient‐physician communication wherein anti‐HIV treatment regimens could increase risks of cardiovascular and renal diseases [27, 28, 29].

Furthermore, the potential relationship between physical indicators, for example sleep and other indicators relevant to mental and social health, has been extensively described in the literature [30, 31, 32]. In Hong Kong, the respondents associated mood with sleep and those in Singapore related mood with medication side effects. This potentially suggested discordance between the two regions wherein the understanding of psychosocial health and its association with physical symptoms could be better among PLHIV in Hong Kong than those in Singapore. Poor quality of sleep, for example sleep disturbances like insomnia or fatigue were observed to be more commonly pronounced among PLHIV who experienced greater psychological distress [32, 33]. The possible lack of awareness of sleep disturbance as a health issue in general and as an HIV‐related health issue could explain for why respondents did not initiate discussions of such issues with their physicians.

Despite many studies demonstrating psychosocial stress as a key factor in lowering immunity and causing inflammation among people living with HIV [34, 35, 36], patients still lacked clarity on the impact and importance of their psychosocial health. Most respondents in our study revealed that they do not actively initiate discussions related to psychosocial health with their physicians. Instead, most PLHIV‐physician dialogues during medical consultation were focused on HIV diagnosis and transmission, treatment acceptance and adherence [37, 38, 39]. Such topics were incidentally more relevant at the initial stages of HIV management. On the contrary, topics more relevant for PLHIV who have achieved virological stability (e.g. sexual health, treatment side‐effects and HIV‐associated stigma [internalized or enacted]) were less discussed [40, 41, 42, 43]. This was supported by this study's findings wherein respondents had perceived their physicians to be more focused on physical care that may be more related to general HIV‐related clinical parameters and medication, while PLHIV themselves would conceal symptoms of HIV and were observed to deprioritize psychosocial health in their self‐care [44]. This suggested a need for better PLHIV‐physician communication focusing on topics such as long‐term health risks, HIV‐associated stigma and interpersonal relationships, as the patient progresses through their HIV journey, that is from the initial diagnosis to reaching virological stability.

Perceived limitations in quality PLHIV‐physician communication due to structural factors associated with a lack of time and/or suitable resources and healthcare system infrastructure were observed in both Singapore and Hong Kong. In Hong Kong, follow‐up HIV consultation sessions were not assigned to the same specialist due to the local policy to rotate specialists; instead, other HCPs like nurses took on the role of developing interpersonal relationships with the patients. Consequently, this may lead to a lower rapport and trust between patients and their physicians and hence, patients are less likely to openly express their condition and related concerns. In Singapore, while the same physician is assigned, consultation dialogues tended to be patient‐initiated and factual as physicians provided clarifications to patients’ queries and concerns. A study suggested that efforts should be focused to improve patient‐physician dialogue quality by aligning on the more salient topics between patients and physicians [41].

However, HIV‐related stigma arising from the individual (internalized stigma), interactions with people (enacted and anticipated stigma) and at healthcare level (structural stigma) could add to PLHIV not being forthcoming to engage openly with physicians and seek psychosocial support, despite the availability of professional psychosocial support services [45, 46, 47, 48]. Negative beliefs and feelings associated with HIV are further exacerbated by PLHIV about themselves, particularly so for women [43, 49–51]. Furthermore, challenges associated with those with greater social constraints as well as group identity (e.g. biological sex and gender identity) could lead minority groups among PLHIV to feel isolated. Heterosexual women living with HIV or with high exposure risk to HIV often shared experiences of stigma due to the connection of HIV to sexual promiscuity [52, 53] which results in a lack of belonging and reluctance to join support groups. This acts as an obstacle to patient empowerment and for support groups to provide a more supportive environment for women.

Non‐binary respondents who have not yet shared their sexuality publicly also had similar concerns of unintended sharing by participating in support groups. In Singapore, before the repeal of the Penal Code Section 377A, men who have sex with men were considered illegal, which could potentially deter them from seeking psychosocial support services in Singapore. Openness to the sharing of HIV status can also be dependent on age as seen in Hong Kong, where it impacts access to psychosocial support services. Supportive services were differently received by PLHIV wherein younger PLHIV had more comfort to seek professional support services like psychiatrists and counsellors, and older PLHIV had a greater preference for social (family and friends) support. Hence, supportive channels must consider the diversity of the HIV community and the intersectionality of challenges anticipated and experienced to allow them to feel empowered to remain connected to the community.

### 4.1 Proposed recommendations

The findings identified opportunities for further patient education, programmes and solutions to improve disease management along their HIV care pathway and the QoL of PLHIV. Challenges remain in the healthcare system which requires support beyond the expertise of an infectious disease specialist or primary care physician to adequately address the multifaceted issues experienced by persons living with HIV. Integration of cross‐disciplinary care services is, therefore, considered essential for PLHIV with prevention and control being achieved through increased awareness, education, early detection and combating HIV‐related issues [54, 55].

Educational efforts to boost the awareness of physical health indicators like sleep, cardiovascular and renal risks as both general and HIV‐related health issues are recommended to help support mental health among patients in both regions. As the cyclic relationship between psychosocial distress and HIV‐related health becomes increasingly apparent, such efforts would help patients better monitor their health indicators and improve their overall wellbeing. Furthermore, initiatives allowing patients to share their concerns prior to their follow‐up consultation appointments could be adopted. This would raise physicians’ awareness and prioritize the patients’ concerns, including but not limited to psychosocial health, rather than the routine dialogue of physical wellbeing. Patient and physician education and training, communication/decision aids for patients could also further facilitate communications and strengthen patient‐physician alliance [41, 56]. The nuances in the understanding of psychosocial health in relation to physical symptoms can inform the need for different engagement approaches for region‐specific patient education.

Multi‐disciplinary expert panels are necessary for the development of a consensus statement on the role of health systems in advancing the long‐term wellbeing of PLHIV from a patient‐centred perspective. Introducing PLHIV to support groups would be beneficial, even for their caregivers to share experiences and information, and offer a support system [49, 53, 55]. Behaviour change techniques such as education, modelling and enablement could mitigate social stigma and internalized stigma [56, 57, 58]. This can strengthen PLHIV's motivation to improve their wellbeing by empowering them to actively participate in decision and self‐care that directly influence their QoL. Training initiatives to equip physicians, other HCPs (e.g. psychologists/psychotherapists, nurses) and personnel from patient support groups with psychosocial counselling skills could support patients to alleviate anxiety during diagnosis and ensure adherence to ART, which in turn improves overall wellbeing. It is also equally important to recruit and equip professionals with the narrative competence to communicate with empathy, build trust and strengthen patient‐physician alliance. This could further empower patients to proactively initiate conversations with their physicians and other HCPs involved in supportive care for an integrated care for their overall wellbeing.

As Singapore and Hong Kong work towards achieving the WHO‐UNAIDs target, there is importance in enforcing multi‐disciplinary integrated HIV care support infrastructure to support the “fourth 90.” Efforts are needed to address and dispel the stigma for PLHIV, strengthen patient‐physician alliance and allow for more integrated healthcare and seamless transition into community care. More integrated and seamless management of patients with other care providers, such as nurses, social workers and community‐based organizations that can support patients in psychosocial support, can ease the burden of physicians managing patients, to achieve the best possible long‐term QoL outcomes for PLHIV.

Overall, this study provided insights into the lived experiences of PLHIV, highlighting the capability, opportunity and motivational barriers. Addressing these behaviour changes for patients and potentially physicians can improve the management of PLHIV's health holistically, empower PLHIV, improve clinical practices and advocate for greater support from other healthcare expertise and community‐based organizations.

### 4.2 Study limitations

There are limitations to the study. This exploratory study was designed to elucidate key themes associated with HIV care among PLHIV who were recruited through local patient support groups. Furthermore, these respondents might have received support from these organizations at any point in their condition, potentially influencing the development of their wellbeing. As such, the impact of HIV on the overall wellbeing of respondents who did not have access to or could not be accessed by patient support organizations could be under‐represented. The study was focused on two higher‐income regions in Asia and consequently, the generalizability to low‐middle income countries in the region may be limited. While the insights from this study may not be generalizable to the larger PLHIV community in Hong Kong and Singapore, the study findings can support future quantitative studies investigating HIV‐related care among larger study populations.

The small sample size of the female heterosexual respondents in this study could be due to recruitment barriers associated with the sensitive nature of HIV [59], thus limiting the study findings towards a male/men who have sex with men‐dominated manner. Future studies are warranted to better understand the impact of living with HIV may have on female patients to provide quality support, especially in the aspects of psychosocial support, to boost the wellbeing of this population. Participation was further limited among transgendered people with HIV who have even greater vulnerability to HIV due to greater stigma and discrimination based on gender identity [60]. Furthermore, the perceptions of physicians and HCPs involved in HIV care, which could shed greater insights on the barriers of holistic wellbeing among PLHIV, were not explored in this study and warrant further investigation.

## 5 CONCLUSIONS

Inadequacies in patient‐physician communications and alliance, and limited focus on psychosocial wellness impact the holistic management of HIV and ultimately treatment outcomes. There is a need in Singapore and Hong Kong to empower PLHIV to establish better communications with their physicians and be more involved in their treatment journey and equally prioritizing their psychosocial wellbeing. Patients would also need to enhance their awareness and knowledge on HIV‐related health issues, boost pro‐health behaviours and actively engage with other HCPs or community‐based organizations for support to improve their overall wellbeing.

## COMPETING INTERESTS

AL and RF are employees of Gilead Sciences, Hong Kong. RG and YJY are employees of Oracle Life Sciences, Singapore (formerly known as Cerner Enviza, Singapore). AC and KLL were representatives of AIDS Concern, Hong Kong during the conduct of the study. The other authors declare that they have no competing interests.

## AUTHORS’ CONTRIBUTIONS

CSW led, and all authors (CSW, AC, KLL, PKHM, SB, VH, YL, RG, YJY, RF and AL) contributed to the conceptualization or design, analyses, or interpretation of data, drafting of the article and critical revision for important intellectual content. YY contributed to the execution, acquisition of data and led manuscript writing. All authors (CSW, AC, KLL, PKHM, SB, VH, YL, RG, YJY, RF and AL) contributed and provided input at the writing stage and the final draft. All authors (CSW, AC, KLL, PKHM, SB, VH, YL, RG, YJY, RF and AL) have read and approved the final manuscript.

## FUNDING

This study was funded by Gilead Sciences, Hong Kong.

## DISCLAIMER

The study (#21‐CERN‐101) was reviewed for exemption by the Pearl Institutional Review Board (IRB) and determined to be exempt from IRB review according to FDA 21 CFR 56.104 and 45CFR46.104(b)(2): (2) Tests, Survey, Interviews for the periods the data are used in the current study. All respondents who participated in the study provided their informed consent.

## Data Availability

The full data that support the findings of this study are available from the corresponding author upon reasonable request.

## References

[1] World Health Organization . Prevalence of HIV among adults aged 15 to 49 (%) [Internet]. [cited 2024 Jan 10]. Available from: https://www.who.int/data/gho/data/indicators/indicator‐details/GHO/prevalence‐of‐hiv‐among‐adults‐aged‐15‐to‐49‐(‐)

[2] Ministry of Health, Singapore . Update on the HIV/AIDS situation in Singapore 2022 (June 2023) [Internet]. 2023 [cited 2024 Jan 10]. Available from: https://www.moh.gov.sg/resources‐statistics/infectious‐disease‐statistics/hiv‐stats/update‐on‐the‐hiv‐aids‐situation‐in‐singapore‐2022‐(june‐2023)

[3] Department of Health Hong Kong . Virtual AIDS Office [Internet]. [cited 2022 Nov 3]. Available from: https://www.aids.gov.hk/english/index.html

[4] Frescura L , Godfrey‐Faussett P , Feizzadeh AA , El‐Sadr W , Syarif O , Ghys PD , et al. Achieving the 95 95 95 targets for all: a pathway to ending AIDS. PLoS One. 2022;17(8):e0272405. 35925943 10.1371/journal.pone.0272405 PMC9352102

[5] Virtual AIDS Office of Hong Kong . Factsheet—HIV/AIDS situation in Hong Kong [2021]. Available from: https://www.aids.gov.hk/english/surveillance/sur_report/hiv_fc2021e.pdf. Accessed 30 August 2023.

[6] National Center for Infectious Diseases . NHIVP Guidance Documents—National Centre for Infectious Diseases [Internet]. [cited 2023 Jan 17]. Available from: https://www.ncid.sg/About‐NCID/OurDepartments/Pages/NHIVP‐Guidance‐Documents.aspx

[7] National Centre for Infectious Diseases . National HIV Programme—National Centre for Infectious Diseases [Internet]. [cited 2022 Nov 4]. Available from: https://www.ncid.sg/About‐NCID/OurDepartments/Pages/National‐HIV‐Programme.aspx

[8] Tan RKJ , Cook AR . Singapore's HIV disclosure law in the context of progress towards the 90‐90‐90 goals: a call for greater action in the Western Pacific. Lancet Reg Health—West Pac. 2022;29:100588. 36106134 10.1016/j.lanwpc.2022.100588 PMC9464955

[9] Hong Kong Advisory Council on AIDS . Recommended HIV/AIDS Strategies for Hong Kong (2022–2027) Consultation Document (Draft of Strategies).

[10] Special Preventive Programme Centre for Health Protection, Department of Health . The Government of the Hong Kong Special Administrative Region. Factsheet on A Feasibility Study of Using a Web‐based Ordering and Result Upload of HIV Self‐testing (HIVST) among Men Who Have Sex with Men (MSM) in Hong Kong [Internet]. 2020. Available from: https://www.aids.gov.hk/pdf/HIVST_eng.pdf

[11] Lau JTF , Tsui HY . Discriminatory attitudes towards people living with HIV/AIDS and associated factors: a population based study in the Chinese general population. Sex Transm Infect. 2005;81(2):113–119. 15800086 10.1136/sti.2004.011767 PMC1764671

[12] Liu H , Hu Z , Li X , Stanton B , Naar‐King S , Yang H . Understanding interrelationships among HIV‐related stigma, concern about HIV infection, and intent to disclose HIV serostatus: a pretest‐posttest study in a rural area of eastern China. AIDS Patient Care STDs. 2006;20(2):133–142. 16475894 10.1089/apc.2006.20.133

[13] Lazarus JV , Safreed‐Harmon K , Barton SE , Costagliola D , Dedes N , del Amo Valero J , et al. Beyond viral suppression of HIV—the new quality of life frontier. BMC Med. 2016;14(1):94. 27334606 10.1186/s12916-016-0640-4 PMC4916540

[14] Costagliola D . Demographics of HIV and aging. Curr Opin HIV AIDS. 2014;9(4):294–301. 24824889 10.1097/COH.0000000000000076

[15] Miners A , Phillips A , Kreif N , Rodger A , Speakman A , Fisher M , et al. Health‐related quality‐of‐life of people with HIV in the era of combination antiretroviral treatment: a cross‐sectional comparison with the general population. Lancet HIV. 2014;1(1):e32–e40. 26423814 10.1016/S2352-3018(14)70018-9

[16] Action for AIDS Singapore . Community Blueprint to End HIV and AIDS [Internet]. [cited 2023 Jan 17]. Available from: https://afa.org.sg/, https://afa.org.sg/

[17] Chan RCH , Mak WWS . Cognitive, regulatory, and interpersonal mechanisms of HIV stigma on the mental and social health of men who have sex with men living with HIV. Am J Mens Health. 2019;13(5):1557988319873778. 31690214 10.1177/1557988319873778 PMC6728686

[18] Yu CH , Huang CY , Ko NY , Tung HH , Huang HM , Cheng SF . The lived experiences of stigmatization in the process of HIV status disclosure among people living with HIV in Taiwan. Int J Environ Res Public Health. 2021;18(10):5089. 34064970 10.3390/ijerph18105089 PMC8150537

[19] Kipke MD , Kubicek K , Weiss G , Wong C , Lopez D , Iverson E , et al. The health and health behaviors of young men who have sex with men. J Adolesc Health. 2007;40(4):342–350. 17367727 10.1016/j.jadohealth.2006.10.019 PMC2955360

[20] Michie S , van Stralen MM , West R . The behaviour change wheel: a new method for characterising and designing behaviour change interventions. Implement Sci. 2011;6(1):42. 21513547 10.1186/1748-5908-6-42 PMC3096582

[21] Oppenheim AN . Questionnaire design and attitude measurement. Basic Books; 1966.

[22] Ideal salary for living in Singapore is S$6,000: survey [Internet]. [cited 2022 Nov 17]. Available from: https://sg.finance.yahoo.com/news/ideal‐salary‐for‐living‐in‐singapore‐is‐s‐6‐000–survey‐074615934.html

[23] Doctor T . What is the average salary in Hong Kong for 2022? [Internet]. Biz 3.0. 2022. [cited 2022 Nov 17]. Available from: https://biz30.timedoctor.com/average‐salary‐in‐hong‐kong/

[24] Boucher LM , O'Brien KK , Baxter LN , Fitzgerald ML , Liddy CE , Kendall CE . Healthy aging with HIV: the role of self‐management support. Patient Educ Couns. 2019;102(8):1565–1569. 30827568 10.1016/j.pec.2019.02.019

[25] Capili B , Anastasi JK , Chang M , Ogedegbe O . Barriers and facilitators to engagement in lifestyle interventions among individuals with HIV. J Assoc Nurses AIDS Care. 2014;25(5):450–457. 24630628 10.1016/j.jana.2014.01.003 PMC4130780

[26] Khoshtarash M , Farahani M , Zareiyan A . Health‐related lifestyle in HIV/AIDS patients: a hybrid concept analysis. HIV AIDS Rev. 2019;18(2):120–130.

[27] Joshi K , Boettiger D , Kerr S , Nishijima T , Van Nguyen K , Ly PS , et al. Changes in renal function with long‐term exposure to antiretroviral therapy in HIV‐infected adults in Asia. Pharmacoepidemiol Drug Saf. 2018;27(11):1209–1216. 30246898 10.1002/pds.4657 PMC6218305

[28] Bijker R , Kumarasamy N , Kiertiburanakul S , Pujari S , Lam W , Chaiwarith R , et al. Cardiovascular disease incidence projections in the TREAT Asia HIV Observational Database (TAHOD). Antivir Ther. 2019;24(4):271–279. 30833516 10.3851/IMP3298 PMC6728217

[29] Shah ASV , Stelzle D , Lee KK , Beck EJ , Alam S , Clifford S , et al. Global burden of atherosclerotic cardiovascular disease in people living with HIV. Circulation. 2018;138(11):1100–1112. 29967196 10.1161/CIRCULATIONAHA.117.033369 PMC6221183

[30] Remien RH , Stirratt MJ , Nguyen N , Robbins RN , Pala AN , Mellins CA . Mental health and HIV/AIDS: the need for an integrated response. AIDS. 2019;33(9):1411–1420. 30950883 10.1097/QAD.0000000000002227 PMC6635049

[31] Cruess DG , Antoni MH , Gonzalez J , Fletcher MA , Klimas N , Duran R , et al. Sleep disturbance mediates the association between psychological distress and immune status among HIV‐positive men and women on combination antiretroviral therapy. J Psychosom Res. 2003;54(3):185–189. 12614827 10.1016/s0022-3999(02)00501-9

[32] Wu J , Wu H , Lu C , Guo L , Li P . Self‐reported sleep disturbances in HIV‐infected people: a meta‐analysis of prevalence and moderators. Sleep Med. 2015;16(8):901–907. 26188954 10.1016/j.sleep.2015.03.027

[33] Huang X , Li H , Meyers K , Xia W , Meng Z , Li C , et al. Burden of sleep disturbances and associated risk factors: a cross‐sectional survey among HIV‐infected persons on antiretroviral therapy across China. Sci Rep. 2017;7(1):3657. 28623361 10.1038/s41598-017-03968-3 PMC5473875

[34] Kołodziej J . Effects of stress on HIV infection progression. HIV AIDS Rev. 2016;15(1):13–16.

[35] Rendina HJ , Weaver L , Millar BM , López‐Matos J , Parsons JT . Psychosocial well‐being and HIV‐related immune health outcomes among HIV‐positive older adults: support for a biopsychosocial model of HIV stigma and health. J Int Assoc Provid AIDS Care. 2019;18:2325958219888462. 31795813 10.1177/2325958219888462 PMC6893929

[36] Chida Y , Vedhara K . Adverse psychosocial factors predict poorer prognosis in HIV disease: a meta‐analytic review of prospective investigations. Brain Behav Immun. 2009;23(4):434–445. 19486650 10.1016/j.bbi.2009.01.013

[37] Laws MB , Beach MC , Lee Y , Rogers WH , Saha S , Korthuis PT , et al. Provider‐patient adherence dialogue in HIV care: results of a multisite study. AIDS Behav. 2013;17(1):148–159. 22290609 10.1007/s10461-012-0143-z PMC3668314

[38] Schneider J , Kaplan SH , Greenfield S , Li W , Wilson IB . Better physician−patient relationships are associated with higher reported adherence to antiretroviral therapy in patients with HIV infection. J Gen Intern Med. 2004;19(11):1096–1103. 15566438 10.1111/j.1525-1497.2004.30418.x PMC1494791

[39] Barfod TS , Hecht FM , Rubow C , Gerstoft J . Physicians’ communication with patients about adherence to HIV medication in San Francisco and Copenhagen: a qualitative study using Grounded Theory. BMC Health Serv Res. 2006;6(1):154. 17144910 10.1186/1472-6963-6-154 PMC1702356

[40] Ports KA , Barnack‐Tavlaris JL , Syme ML , Perera RA , Lafata JE . Sexual health discussions with older adult patients during periodic health exams. J Sex Med. 2014;11(4):901–908. 24517714 10.1111/jsm.12448 PMC4657130

[41] Laws MB , Epstein L , Lee Y , Rogers W , Beach MC , Wilson IB . The association of visit length and measures of patient‐centered communication in HIV care: a mixed methods study. Patient Educ Couns. 2011;85(3):e183–e188. 21592716 10.1016/j.pec.2011.04.013 PMC3158953

[42] Epstein RM , Morse DS , Frankel RM , Frarey L , Anderson K , Beckman HB . Awkward moments in patient‐physician communication about HIV risk. Ann Intern Med. 1998;128(6):435–442. 9499326 10.7326/0003-4819-128-6-199803150-00003

[43] Malta M , Todd CS , Stibich MA , Garcia T , Pacheco D , Bastos FI . Patient–provider communication and reproductive health among HIV‐positive women in Rio de Janeiro, Brazil. Patient Educ Couns. 2010;81(3):476–482. 20947284 10.1016/j.pec.2010.09.013

[44] Balbin EG , Ironson GH , Solomon GF . Stress and coping: the psychoneuroimmunology of HIV/AIDS. Best Pract Res Clin Endocrinol Metab. 1999;13(4):615–633. 10.1053/beem.1999.0047 10903818

[45] Chan RCH , Mak WWS , Ma GYK , Cheung M . Interpersonal and intrapersonal manifestations of HIV stigma and their impacts on psychological distress and life satisfaction among people living with HIV: toward a dual‐process model. Qual Life Res. 2021;30(1):145–156. 32909160 10.1007/s11136-020-02618-y

[46] Reinius M , Zeluf Andersson G , Svedhem V , Wettergren L , Wiklander M , Eriksson LE . Towards a new understanding of HIV‐related stigma in the era of efficient treatment–a qualitative reconceptualization of existing theory. J Adv Nurs. 2021;77(5):2472–2480. 33599309 10.1111/jan.14774

[47] Yu F , Hsiao YH , Park S , Kambara K , Allan B , Brough G , et al. The influence of anticipated HIV stigma on health‐related behaviors, self‐rated health, and treatment preferences among people living with HIV in East Asia. AIDS Behav. 2023;27(4):1287‐1303. 36348191 10.1007/s10461-022-03865-5 PMC10036452

[48] Gagnon M . Re‐thinking HIV‐related stigma in health care settings: a qualitative study. J Assoc Nurses AIDS Care. 2015;26(6):703–719. 26300466 10.1016/j.jana.2015.07.005

[49] Fauk NK , Mwanri L , Hawke K , Mohammadi L , Ward PR . Psychological and social impact of HIV on women living with HIV and their families in low‐ and middle‐income Asian countries: a systematic search and critical review. Int J Environ Res Public Health. 2022;19(11):6668. 35682255 10.3390/ijerph19116668 PMC9180788

[50] Fauk NK , Hawke K , Mwanri L , Ward PR . Stigma and discrimination towards people living with HIV in the context of families, communities, and healthcare settings: a qualitative study in Indonesia. Int J Environ Res Public Health. 2021;18(10):5424. 34069471 10.3390/ijerph18105424 PMC8159085

[51] Manopaiboon C , Shaffer N , Clark L , Bhadrakom C , Siriwasin W , Chearskul S , et al. Impact of HIV on families of HIV‐infected women who have recently given birth, Bangkok, Thailand. J Acquir Immune Defic Syndr Hum Retrovirol. 1998;18(1):54–63. 9593459 10.1097/00042560-199805010-00009

[52] Chan KY , Rungpueng A , Reidpath DD . AIDS and the stigma of sexual promiscuity: Thai nurses’ risk perceptions of occupational exposure to HIV. Cult Health Sex. 2009;11(4):353–368. 19263260 10.1080/13691050802621161

[53] Paudel V , Baral KP . Women living with HIV/AIDS (WLHA), battling stigma, discrimination and denial and the role of support groups as a coping strategy: a review of literature. Reprod Health. 2015;12:53. 26032304 10.1186/s12978-015-0032-9 PMC4467680

[54] Elgalib A , Al‐Sawafi H , Kamble B , Al‐Harthy S , Al‐Sariri Q . Multidisciplinary care model for HIV improves treatment outcome: a single‐centre experience from the Middle East. AIDS Care. 2018;30(9):1114–1119. 29792340 10.1080/09540121.2018.1479028

[55] Sherer R , Stieglitz K , Narra J , Jasek J , Green L , Moore B , et al. HIV multidisciplinary teams work: support services improve access to and retention in HIV primary care. AIDS Care. 2002;14(sup1):31–44. 10.1080/09540120220149975 12204140

[56] Integration of Mental Health and HIV Interventions . Key considerations. Geneva: Joint United Nations Programme on HIV/AIDS and the World Health Organization; 2022.

[57] Li L , Lee SJ , Jiraphongsa C , Khumtong S , Iamsirithaworn S , Thammawijaya P , et al. Improving the health and mental health of people living with HIV/AIDS: 12‐month assessment of a behavioral intervention in Thailand. Am J Public Health. 2010;100(12):2418–2425. 20966372 10.2105/AJPH.2009.185462 PMC2978166

[58] Andersson GZ , Reinius M , Eriksson LE , Svedhem V , Esfahani FM , Deuba K , et al. Stigma reduction interventions in people living with HIV to improve health‐related quality of life. Lancet HIV. 2020;7(2):e129–e140. 31776098 10.1016/S2352-3018(19)30343-1 PMC7343253

[59] Remien RH , Chowdhury J , Mokhbat JE , Soliman C , Adawy ME , El‐Sadr W . Gender and care: access to HIV testing, care and treatment. J Acquir Immune Defic Syndr. 2009;51(Suppl 3):S106–S110. 19553777 10.1097/QAI.0b013e3181aafd66 PMC2793673

[60] Magno L , da Silva LAV , Veras MA , Pereira‐Santos M , Dourado I . Estigma e discriminação relacionados à identidade de gênero e à vulnerabilidade ao HIV/aids entre mulheres transgênero: revisão sistemática. Cad Saúde Pública. 2019;35:e00112718. 30994744 10.1590/0102-311X00112718

